# Characterization of the chloroplast genome of *Chlorolobion braunii* ITBB-AG6 isolated from a treatment pond of sanitary sewage

**DOI:** 10.1080/23802359.2023.2237687

**Published:** 2023-07-27

**Authors:** Yaojia Mu, Shuai Ma, Deguan Tan, Xuepiao Sun, Jiaming Zhang

**Affiliations:** a College of Agriculture, Hainan University, Haikou, China; b Institute of Tropical Bioscience and Biotechnology, Hainan Key Laboratory of Microbiological Resources, Hainan Bioenergy Center, Chinese Academy of Tropical Agricultural Sciences, Haikou, China

**Keywords:** *Chlorolobion braunii*, chloroplast genome, phylogenetic analysis, terminal inverted repeat

## Abstract

Sphaeropleales is an order of fast-growing microalgae with high oil content and high efficiency in sewage treatment, in which photosynthesis plays a critical role. We isolated a strain of Sphaeropleales, *Chlorolobion braunii* ITBB-AG6 from an azolla community in a sewage pond, and sequenced its chloroplast genome. The complete genome has a length of 154 kb with a GC content of 31.7%. A total of 89 genes were annotated, including 56 protein-coding genes, 30 tRNA genes, and three rRNA genes. Out of the protein coding genes, 64.3% are involved in photosynthesis, 28.6% are involved in protein synthesis, and 7.1% are involved in ATP synthesis. Transfer RNA genes for 20 amino acids were identified, in which tRNA genes for methionine, leucine, and arginine are tripled, whereas tRNA genes for glutamic acid, glycine, serine, and threonine are doubled. Terminal inverted repeats of 27.9 kb containing 10 genes related to photosynthesis and chloroplast division are present in the genome, suggesting that photosynthesis was strengthened in the evolutionary history. Phylogenetic analysis indicates that *C. braunii* ITBB-AG6 falls in the family Selenastraceae and is most closely related to *Monoraphidium neglectum.*

## Introduction

Sphaeropleales is one of the most important orders in the class Chlorophyceae. It contains some common freshwater species (Fucikova et al. 2014), such as *Ankistrodesmus falcatus* (Corda) Ralfs, 1848 (Wang et al. 2020), *Chlorolobion braunii* (Naegeli) Komarek (Baracho et al. 2019), and *Monoraphidium braunii* (Nägeli ex Kützing) Komárková-Legnerová 1969 (Gattullo et al. 2012). These species have been used in applications such as bioassays, bioremediation, and biofuel production (Gorman and Levine 1965; Wang et al. 2020; El-Sheekh et al. 2023). However, Sphaeropleales is relatively poorly understood in terms of diversity and evolution, compared to its sister order Volvocales, which contains the versatile model species *Chlamydomonas reinhardtii*, and is often used in the investigation of the evolution of multicellularity (Fucikova et al. 2016). Chloroplast genome data are widely used to infer phylogenetic relationships of plants and algae, and complex patterns of sequence evolution were revealed in Sphaeropleales (Fucikova et al. 2016). This study reports the chloroplast genome of a Sphaeropleales species, *C. braunii* ITBB-AG6.

## Materials

**Figure 1.**
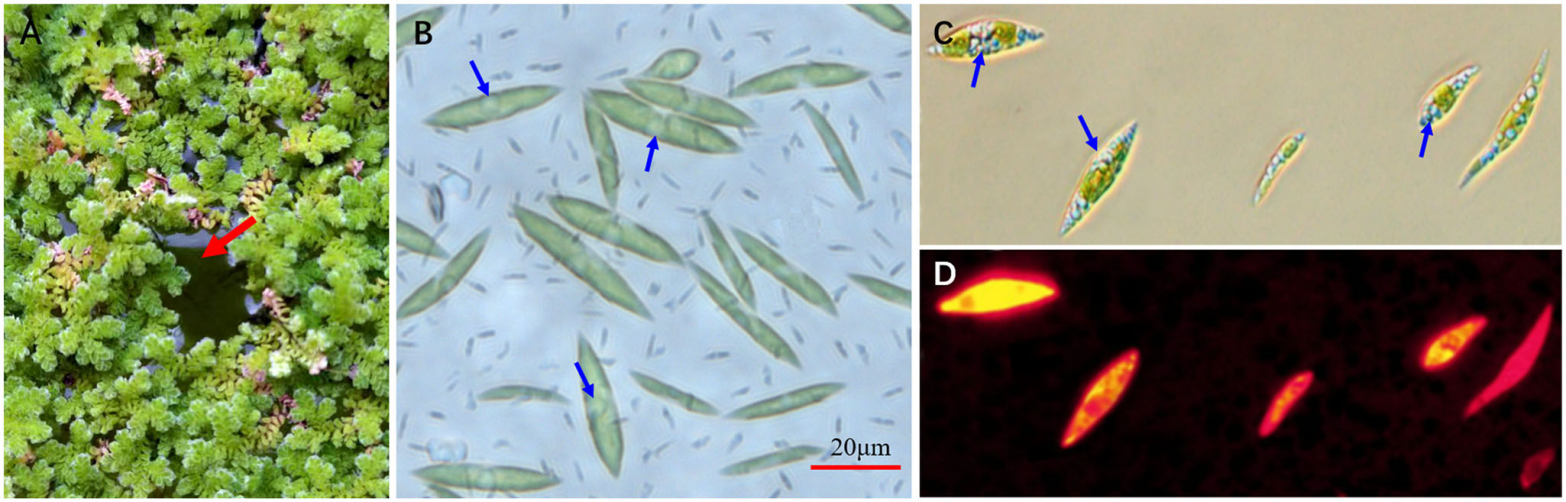
The azolla-algae community and the algal cells. (A) Azolla-algae community; (B) wild algal cells from the community; algal cells grown in TAP medium and stained with Nile red, and observed under bright (C) and fluorescent light (D). Red arrow indicates the algal film in the gap between azolla plants; blue arrows indicate the oil droplets in the algal cells. (A, B) Taken by Jiaming Zhang; (C, D) taken by Yaojia Mu.

*C. braunii* strain ITBB-AG6 was isolated from an azolla community in a sewage treatment pond operated by Jiaming Zhang’s laboratory at the experimental station of the Institute of Tropical Bioscience and Biotechnology, CATAS in Danzhou City, Hainan Province, China (19.5211N, 109.5119E). Classification of the strain was performed by referring to the images of the type strains in the SAG algal stocks (https://www.uni-goettingen.de/en/184982.html) and the AlgaeBase (Guiry and Guiry 2018) (Figure 1). For DNA isolation, the strain was cultured in TAP medium (Gorman and Levine 1965) and centrifuged to harvest the cells. A sample of the culture is stored at the ClonBank of the Institute of Tropical Bioscience and Biotechnology at −80 °C in 15% glycerol with the voucher number ITBB-AG6 (Curator, Deguan Tan, tandeguan@itbb.org.cn).

## Methods

For fluorescence microscopy, algal cells were stained with Nile red (Chen et al. 2009), and observed under a fluorescent microscope (Olympus IX73, Shinjuku City, Japan) with an excitation wavelength of 530 nm.

Genomic DNA was extracted using a Universal Genomic DNA Extraction Kit (Sangon, Shanghai, China) according to the manufacturer’s instruction. The genome was sequenced using Illumina Hiseq 2500 and Nanopore platforms, and was assembled with Canu v1.5 (Koren et al. 2017) and wtdbg2 (Ruan and Li 2020). The scaffold containing the chloroplast genome was identified by a local blast search using a chloroplast sequence of *Chlorella vulgaris* (MT920676.1) as a reference (Han et al. 2021). The overlapped sequence in the 5′ and 3′ ends of the scaffold was removed with MacVector 13.6. The assembly quality was assessed by mapping the Illumina reads to the assembly, followed by calculating the sequence depth and coverage using a recently published protocol (Ni et al. 2023). The protein coding genes were annotated with GeSeq webServer (https://chlorobox.mpimp-golm.mpg.de/geseq.html, as well as by sequence alignment with MacVector 13.6). Transfer RNA genes were annotated with tRNAscan-SE (Chan and Lowe 2019). The circular genome was visualized with MacVector 13.6.

For phylogenetic analysis, chloroplast genomes of 20 algal species from Sphaeropleales were retrieved from GenBank. Coding DNA sequences of 20 genes (*atpB*, *atpE*, *atpF*, *psbZ*, *psbE*, *psbA*, *rbcL*, *rps4*, *rps19*, *rps12*, *rpl2*, *rpl36*, *rpl5*, *ycf3*, *ycf4*, *psaB*, *psaC*, *petD*, *petG*, and *petA*) that were shared by all taxa were extracted, translated, and combined in the same order. The amino acid sequences were aligned with Clustal Omega (Sievers and Higgins 2021). Phylogenetic trees were inferred by using the maximum-likelihood (ML) methods with 1000 bootstrap replicates in MEGAX (Kumar et al. 2018). The tree was rooted with a *Chlorella vulgaris* genome (Han et al. 2021). The evolutionary history was inferred using the JTT matrix-based model (Jones et al. 1992). The tree with the highest log likelihood (–43849.76) is shown. The percentage of trees in which the associated taxa clustered together is shown next to the branches.

## Results

**Figure 2.**
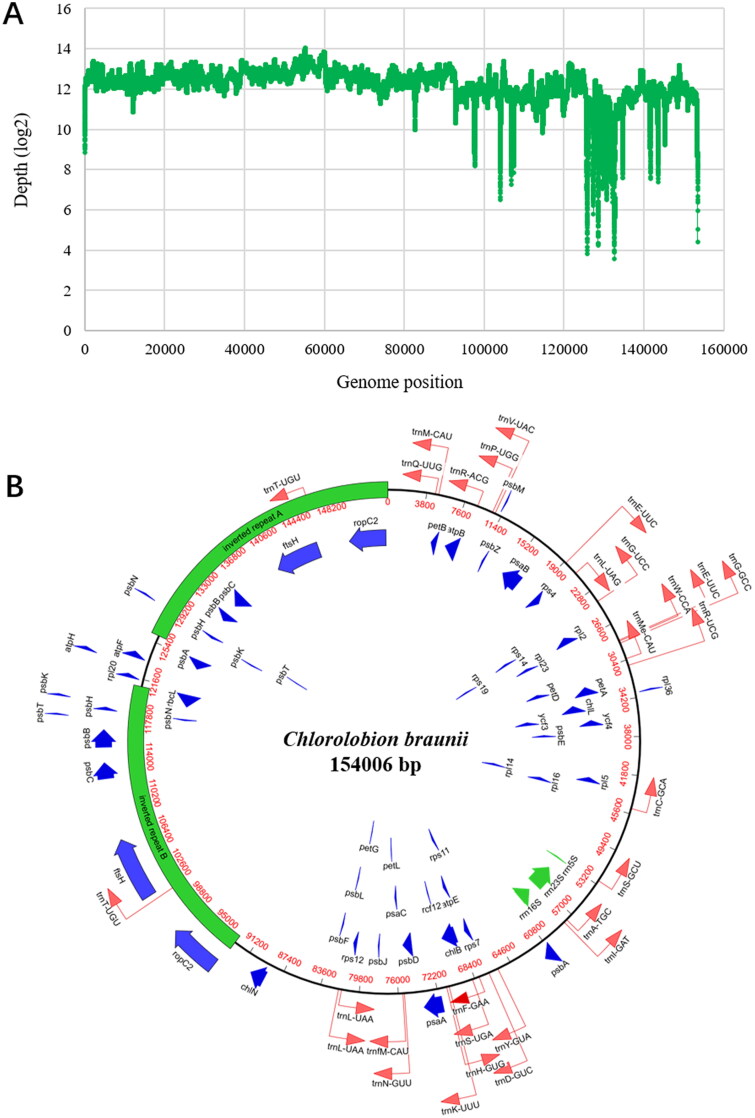
Organization of the chloroplast genome of *C. braunii* ITBB-AG6. (A) Coverage depth plot of the genome, the depth was normalized using the logarithm based on 2. (B) Circular map of the genome with genes’ location, size, and orientation indicated by blue (CDS), green (rRNA), and red (tRNA) arrows.

The chloroplast genome of *C. braunii* ITBB-AG6 has a length of 154,006 bp with a GC content of 31.7%, which is similar to the chloroplast genome of *M. neglectum* (NW_014013626, 32.4%). The average sequence depth is 5383× with a few nucleotides having a low coverage depth of tens (Figure 2(A)) due to uneven allocation of reads to repetitive fragments (see below).

A total of 89 genes were annotated, including 56 protein-coding genes, 30 tRNA genes, and three rRNA genes (Table 1). Out of the protein coding genes, 64.3% (36/56) are involved in photosynthesis (Table 1), 28.6% (16/56) are involved in protein synthesis, and 7.1% (4/56) are involved in ATP synthesis. The three ribosomal RNA genes (*rrn5S*, *rrn16S*, *rrn23S*) are not interrupted by introns as observed in its mitogenomes (unpublished data). Transfer RNA genes for 20 amino acids were identified, in which the tRNA genes for methionine, leucine, and arginine are tripled, whereas the tRNA genes for glutamic acid, glycine, serine, and threonine are doubled (Table 1). Terminal inverted repeats with a length of 27.9 kb and identities of 99.6% were identified (Figure 2(B)). Ten genes (*ropC2*, *ftsH*, *psbC*, *psbB*, *psbT*, *psbH*, *psbK*, *psbN*, *rbcL*, and *trnT*) most related to photosynthesis and chloroplast division are located in the repeats, suggesting that photosynthesis may have been strengthened in the evolution of this strain.

**Table 1.** Gene list of the chloroplast genome of *C. braunii* ITBB-AG6.

Gene	Type	Position (bp)		Chain	Note
		From	To		
petB	CDS	5214	5861	H	Cytochrome b6 of cytochrome b6/f complex
atpB	CDS	6961	8406	H	CF1 beta subunit of ATP synthase;
psbM	CDS	11192	11299	L	Protein M of photosystem II
psbZ	CDS	11751	11939	H	Protein Z of photosystem II
psaB	CDS	14630	16837	H	Apoprotein A2 of photosystem I
rps4	CDS	19557	20333	L	Ribosomal protein S4
rps14	CDS	24207	24509	H	Ribosomal protein S14
rps19	CDS	24667	24948	H	Ribosomal protein S19
rpl2	CDS	25446	26273	H	Ribosomal protein L2
rpl23	CDS	26677	27039	H	Ribosomal protein L23
petD	CDS	31117	31599	H	Subunit IV of cytochrome b6/f complex
petA	CDS	31904	32875	H	Apocytochrome f of cytochrome b6/f complex
rpl36	CDS	33578	33691	H	Ribosomal protein L36
chlL	CDS	33861	34742	H	ATP-binding subunit of protochlorophyllide reductase
ycf3	CDS	35172	35684	H	Hypothetical chloroplast RF3
ycf4	CDS	35936	36571	H	Hypothetical chloroplast RF4
psbE	CDS	37518	37763	H	Cytochrome b559 alpha subunit of photosystem II
rpl5	CDS	42798	43340	H	Ribosomal protein L5
rpl14	CDS	43453	43818	H	Ribosomal protein L14
rpl16	CDS	44132	44542	H	Ribosomal protein L16
psbA	CDS	60554	61615	L	D1 reaction center protein of photosystem II
rps11	CDS	66259	66651	L	Ribosomal protein S11
rps7	CDS	67085	67591	L	Ribosomal protein S7
atpE	CDS	67719	68129	L	CF1 epsilon subunit of ATP synthase
chlB	CDS	68765	70453	L	ChlB subunit of protochlorophyllide reductase
rcf12	CDS	70670	70771	L	Chloroplast protein RF12
psaA	CDS	71938	73677	L	P700 apoprotein A1 of photosystem I
psbD	CDS	74155	75216	L	D2 reaction center protein of photosystem II
psaC	CDS	75690	75935	L	Subunit VII of photosystem I (Fe-S polypeptide)
petL	CDS	76216	76314	L	Subunit VI of cytochrome b6/f complex
psbJ	CDS	78036	78164	L	J protein of photosystem II
rps12	CDS	80740	81132	L	Ribosomal protein S12
petG	CDS	81195	81308	H	Subunit V of cytochrome b6/f complex
psbL	CDS	82155	82271	H	L protein of photosystem II
psbF	CDS	82833	82967	H	Photosystem II protein VI
chlN	CDS	88811	90157	L	Protochlorophyllide reductase subunit N
rps3	CDS	90524	92590	L	Ribosomal protein S3
ropC2	CDS	93224	97660	L	Beta subunit of RNA polymerase
psbC	CDS	112308	113693	L	Protein C of photosystem II
psbB	CDS	115033	116559	L	Protein B of photosystem II
psbT	CDS	117527	117622	L	T protein T of photosystem II
psbN	CDS	118031	118165	H	N protein of photosystem II
psbH	CDS	118251	118511	L	10 kDa phosphoprotein of photosystem II
psbK	CDS	118943	119083	L	K protein of photosystem II
rbcL	CDS	119677	121104	H	Ribulose-1,5-bisphosphate carboxylase/oxygenase large subunit
rpl20	CDS	121504	121848	L	Ribosomal protein L20
atpH	CDS	122780	123028	L	ATP synthase CF0 C subunit
atpF	CDS	123427	123969	L	CF0 subunit I of ATP synthase
psbA	CDS	124405	125466	H	D1 reaction center protein of photosystem II
psbK	CDS	127942	128082	H	K protein of photosystem II
psbH	CDS	128514	128774	H	10 kDa phosphoprotein of photosystem II
psbN	CDS	128860	128994	L	N protein of photosystem II
psbT	CDS	129400	129495	H	T protein of photosystem II
psbB	CDS	130461	131986	H	CP47 chlorophyll apoprotein of photosystem II
psbC	CDS	133316	134701	H	Protein C of photosystem II
ropC2	CDS	149349	153786	H	Beta subunit of RNA polymerase
repeat B	repeat	92944	120904	H	Terminal inverted repeat B
rrn5S	rRNA	53088	53212	H	5S ribosomal RNA
rrn23S	rRNA	54734	57376	H	23S ribosomal RNA
rrn16S	rRNA	58286	59795	H	16S ribosomal RNA
trnQ-UUG	tRNA	4578	4652	H	tRNA-Gln
trnM-CAU	tRNA	4903	4975	H	tRNA-Met
trnR(acg)	tRNA	8851	8924	H	tRNA-Arg
trnP(ugg)	tRNA	10412	10485	H	tRNA-Pro
trnV(uac)	tRNA	10680	10754	H	tRNA-Val
trnE(uuc)	tRNA	19180	19252	L	tRNA-Glu
trnL-UAG	tRNA	20565	20647	L	tRNA-Leu
trnG-UCC	tRNA	23963	24037	H	tRNA-Gly
trnE(uuc)	tRNA	28269	28342	H	tRNA-Glu
trnW-CCA	tRNA	28377	28449	H	tRNA-Trp
trnG(gcc)	tRNA	28583	28655	H	tRNA-Gly
trnMe(cau)	tRNA	30202	30275	H	tRNA-Met
trnR-UCG	tRNA	30881	30952	H	tRNA-Arg
trnC-GCA	tRNA	45005	45076	H	tRNA-Cys
trnS(gcu)	tRNA	52720	52809	H	tRNA-Ser
trnA(UGC)	tRNA	57629	57701	H	tRNA-Ala
trnI(gat)	tRNA	57969	58045	H	tRNA-Ile
trnY(gua)	tRNA	65955	66036	L	tRNA-Tyr
trnD(guc)	tRNA	66913	66986	L	tRNA-Asp
trnF(gaa)	tRNA	67941	68114	L	tRNA-Phe
trnS(uga)	tRNA	68624	68710	L	tRNA-Ser
trnK(uuu)	tRNA	70998	71069	L	tRNA-Lys
trnH-GUG	tRNA	71168	71240	H	tRNA-His
trnN(guu)	tRNA	75391	75462	L	tRNA-Asn
trnMf(cau)	tRNA	75544	75617	L	tRNA-Met
trnL(uaa)	tRNA	81480	81529	H	trnL-Leu
trnL(uaa)	tRNA	81783	81816	H	tRNA-Leu
trnT(ugu)	tRNA	100884	100958	L	tRNA-Thr
trnT(ugu)	tRNA	146053	146125	H	tRNA-Thr

**Figure 3.**
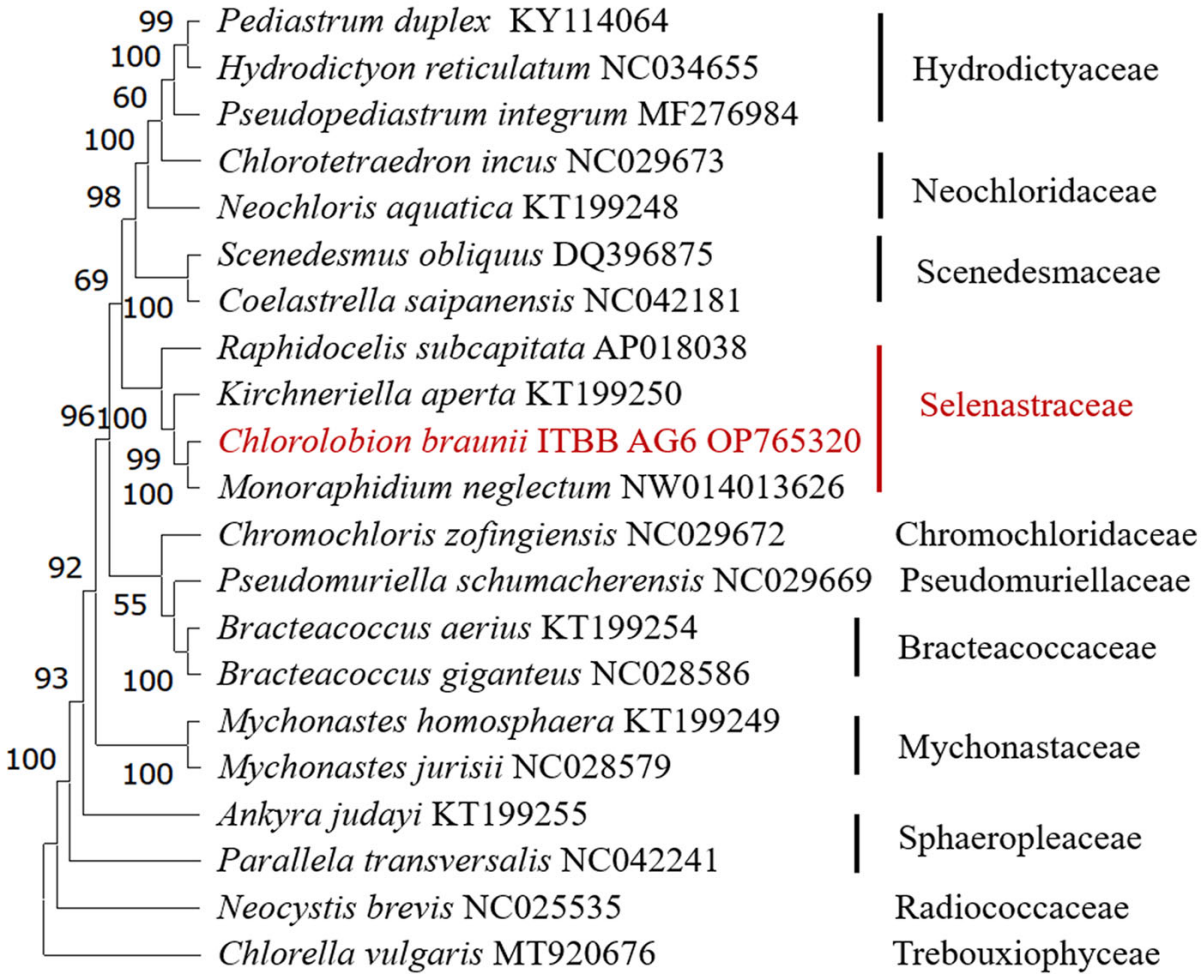
Consensus maximum-likelihood tree of Sphaeropleales (chlorophyta). The tree is rooted with a *Chlorella vulgaris* genome (Han et al. 2021). Bootstrap supports of 1000 replicates are shown above the branches. The strain in this study is shown in red.

Phylogenomic analysis of related species in Sphaeropleales revealed that *C. braunii* ITBB-AG6 is positioned in the family Selenastraceae with 100% bootstrap support, and it is most closely related to *M. neglectum* (Figure 3).

## Discussion and conclusions

*C. braunii* ITBB-AG6 was isolated from a sewage pond covered by azolla for phytoremediation. This pond was once dominated by Trebouxiophyceae algae (e.g. *Chlorella vulgaris*; Hu et al. 2020) before azolla was applied. When the pond was covered by azolla for a few days, ITBB-AG6 became the dominant algal strain, and finally formed a algal film in surface gaps left by azolla (Figure 1), which prevented sunlight from penetration to submerged algae. The competitive advantage of *C. braunii* may have come from its large oil body in the cell (Figure 1), which allowed it to float on water surface and grow in the gaps between azolla plants. The recent duplication of photosystem proteins in its chloroplast genome may have also played a role in enhancing its competitive advantage (Figure 2(B)), which has not been observed in other green algal species.

Phylogenomic analysis using the chloroplast genomes of Sphaeropleales revealed that strain ITBB-AG6 is most closely related to *M. neglectum* (Figure 3), and falls in the family Selenastraceae. Monoraphidium and Chlorolobion are two closely related genera, and are not easy to distinguish by morphology. *C. braunii* was once named as *Monoraphidium braunii* (Nägeli ex Kützing) Komárková-Legnerová (Komárková-Legnerová 1969), but renamed as *Chlorolobion braunii* (Nägeli) Komárek (Komárek 1979). Our phylogenomic analysis supports this nomenclature.

In summary, the chloroplast genome of *C. braunii* ITBB-AG6 was sequenced and annotated, and a novel insight into the competitive advantage of this strain over the other microalgae in the azolla community was revealed. This knowledge may be useful in the bioenergy and sewage treatment industry.

## Data Availability

The complete chloroplast genome sequence is openly available in the GenBank of NCBI at https://www.ncbi.nlm.nih.gov under the accession no. OP765320. The Illumina reads generated by the authors to assemble this genome are available under GenBank BioProject no. PRJNA931121, BioSample no. SAMN33050851, and SRA no. SRR23329251.

## References

[CIT0001] Baracho DH, Silva JC, Lombardi AT. 2019. The effects of copper on photosynthesis and biomolecules yield in *Chlorolobion braunii*. J Phycol. 55(6):1335–1347. doi: 10.1111/jpy.12914. 31408527

[CIT0002] Chan PP, Lowe TM. 2019. tRNAscan-SE: searching for tRNA genes in genomic sequences. Methods Mol Biol. 1962:1–14. doi: 10.1007/978-1-4939-9173-0_1. 31020551 PMC6768409

[CIT0003] Chen W, Zhang C, Song L, Sommerfeld M, Hu Q. 2009. A high throughput Nile red method for quantitative measurement of neutral lipids in microalgae. J Microbiol Methods. 77(1):41–47. doi: 10.1016/j.mimet.2009.01.001. 19162091

[CIT0004] El-Sheekh MM, Galal HR, Mousa ASH, Farghl AAM. 2023. Coupling wastewater treatment, biomass, lipids, and biodiesel production of some green microalgae. Environ Sci Pollut Res Int. 30(12):35492–35504. doi: 10.1007/s11356-023-25628-y. 36735132 PMC10017629

[CIT0005] Fucikova K, Lewis PO, Lewis LA. 2014. Putting incertae sedis taxa in their place: a proposal for ten new families and three new genera in Sphaeropleales (Chlorophyceae, Chlorophyta). J Phycol. 50(1):14–25. doi: 10.1111/jpy.12118. 26988005

[CIT0006] Fucikova K, Lewis PO, Lewis LA. 2016. Chloroplast phylogenomic data from the green algal order Sphaeropleales (Chlorophyceae, Chlorophyta) reveal complex patterns of sequence evolution. Mol Phylogenet Evol. 98:176–183. doi: 10.1016/j.ympev.2016.01.022. 26903036

[CIT0007] Gattullo CE, Bahrs H, Steinberg CE, Loffredo E. 2012. Removal of bisphenol A by the freshwater green alga *Monoraphidium braunii* and the role of natural organic matter. Sci Total Environ. 416:501–506. doi: 10.1016/j.scitotenv.2011.11.033. 22209372

[CIT0008] Gorman DS, Levine RP. 1965. Cytochrome f and plastocyanin: their sequence in the photosynthetic electron transport chain of *Chlamydomonas reinhardi*. Proc Natl Acad Sci U S A. 54(6):1665–1669. doi: 10.1073/pnas.54.6.1665. 4379719 PMC300531

[CIT0009] Guiry MD, Guiry GM. 2018. AlgaeBase.World-wide electronic publication, National University of Ireland, Galway. https://www.algaebase.org; [cited 2023 Mar 2].

[CIT0010] Han B, Mu Y, Tan D, Ma S, Fu L, Sun X, Zhang J. 2021. The chloroplast genome of a unicellular green alga strain isolated from the rubber processing wastewater. Mitochondrial DNA B Resour. 6(1):15–16. doi: 10.1080/23802359.2020.1844090. 33659644 PMC7872553

[CIT0011] Hu X, Tan D, Fu L, Sun X, Zhang J. 2020. Characterization of the mitochondrion genome of a *Chlorella vulgaris* strain isolated from rubber processing wastewater. Mitochondrial DNA B Resour. 5(3):2732–2733. doi: 10.1080/23802359.2020.1789004. 33457925 PMC7782133

[CIT0012] Jones DT, Taylor WR, Thornton JM. 1992. The rapid generation of mutation data matrices from protein sequences. Comput Appl Biosci. 8(3):275–282. doi: 10.1093/bioinformatics/8.3.275. 1633570

[CIT0013] Komárek J. 1979. Änderungen in der Taxonomie der Chlorokokkalalgen. Arch Für Hydrobiol Suppl. 56(24):239–263.

[CIT0014] Komárková-Legnerová J. 1969. The systematics and ontogenesis of the genera Ankistrodesmus Corda and Monoraphidium gen. nov. In: Fott B, editor. Studies in phycology. Prague: Academia Publishing House of the Czechoslovak Academy of Sciences; p. 75–144.

[CIT0015] Koren S, Walenz BP, Berlin K, Miller JR, Bergman NH, Phillippy AM. 2017. Canu: scalable and accurate long-read assembly via adaptive k-mer weighting and repeat separation. Genome Res. 27(5):722–736. doi: 10.1101/gr.215087.116. 28298431 PMC5411767

[CIT0016] Kumar S, Stecher G, Li M, Knyaz C, Tamura K. 2018. MEGA X: molecular evolutionary genetics analysis across computing platforms. Mol Biol Evol. 35(6):1547–1549. doi: 10.1093/molbev/msy096. 29722887 PMC5967553

[CIT0017] Ni Y, Li J, Zhang C, Liu C. 2023. Generating sequencing depth and coverage map for organelle genomes. protocols.io. doi: 10.17504/protocols.io.4r3l27jkxg1y/v1.

[CIT0018] Ruan J, Li H. 2020. Fast and accurate long-read assembly with wtdbg2. Nat Methods. 17(2):155–158. doi: 10.1038/s41592-019-0669-3. 31819265 PMC7004874

[CIT0019] Sievers F, Higgins DG. 2021. The clustal omega multiple alignment package. Methods Mol Biol. 2231:3–16. doi: 10.1007/978-1-0716-1036-7_1. 33289883

[CIT0020] Wang Q, Shen Q, Wang J, Zhang Y, Zhang Z, Lei Z, Shimizu K, Lee DJ. 2020. Fast cultivation and harvesting of oil-producing microalgae *Ankistrodesmus falcatus var. acicularis* fed with anaerobic digestion liquor via biogranulation in addition to nutrients removal. Sci Total Environ. 741:140183. doi: 10.1016/j.scitotenv.2020.140183. 32563780

